# Omega-9 Oleic Acid, the Main Compound of Olive Oil, Mitigates Inflammation during Experimental Sepsis

**DOI:** 10.1155/2018/6053492

**Published:** 2018-11-13

**Authors:** Isabel Matos Medeiros-de-Moraes, Cassiano Felippe Gonçalves-de-Albuquerque, Angela R. M. Kurz, Flora Magno de Jesus Oliveira, Victor Hugo Pereira de Abreu, Rafael Carvalho Torres, Vinícius Frias Carvalho, Vanessa Estato, Patrícia Torres Bozza, Markus Sperandio, Hugo Caire de Castro-Faria-Neto, Adriana Ribeiro Silva

**Affiliations:** ^1^Laboratório de Imunofarmacologia, Instituto Oswaldo Cruz, FIOCRUZ, Rio de Janeiro, RJ, Brazil; ^2^Laboratório de Imunofarmacologia, Instituto Biomédico, Universidade Federal do Estado do Rio de Janeiro, Rio de Janeiro, RJ, Brazil; ^3^Walter Brendel Centre, Ludwig Maximilians University München, Klinikum der Universität, Germany; ^4^The Centenary Institute, Camperdown, New South Wales, Australia; ^5^Laboratório de Inflamação, Instituto Oswaldo Cruz, FIOCRUZ, Rio de Janeiro, RJ, Brazil; ^6^German Center for Cardiovascular Research (DZHK) Partner Site, Munich Heart Alliance, Munich, Germany; ^7^Universidade Estácio de Sá, Programa de Produtividade Científica, Rio de Janeiro, RJ, Brazil

## Abstract

The Mediterranean diet, rich in olive oil, is beneficial, reducing the risk of cardiovascular diseases and cancer. Olive oil is mostly composed of the monounsaturated fatty acid omega-9. We showed omega-9 protects septic mice modulating lipid metabolism. Sepsis is initiated by the host response to infection with organ damage, increased plasma free fatty acids, high levels of cortisol, massive cytokine production, leukocyte activation, and endothelial dysfunction. We aimed to analyze the effect of omega-9 supplementation on corticosteroid unbalance, inflammation, bacterial elimination, and peroxisome proliferator-activated receptor (PPAR) gamma expression, an omega-9 receptor and inflammatory modulator. We treated mice for 14 days with omega-9 and induced sepsis by cecal ligation and puncture (CLP). We measured systemic corticosterone levels, cytokine production, leukocyte and bacterial counts in the peritoneum, and the expression of PPAR gamma in both liver and adipose tissues during experimental sepsis. We further studied omega-9 effects on leukocyte rolling in mouse cremaster muscle-inflamed postcapillary venules and in the cerebral microcirculation of septic mice. Here, we demonstrate that omega-9 treatment is associated with increased levels of the anti-inflammatory cytokine IL-10 and decreased levels of the proinflammatory cytokines TNF-*α* and IL-1*β* in peritoneal lavage fluid of mice with sepsis. Omega-9 treatment also decreased systemic corticosterone levels. Neutrophil migration from circulation to the peritoneal cavity and leukocyte rolling on the endothelium were decreased by omega-9 treatment. Omega-9 also decreased bacterial load in the peritoneal lavage and restored liver and adipose tissue PPAR gamma expression in septic animals. Our data suggest a beneficial anti-inflammatory role of omega-9 in sepsis, mitigating leukocyte rolling and leukocyte influx, balancing cytokine production, and controlling bacterial growth possibly through a PPAR gamma expression-dependent mechanism. The significant reduction of inflammation detected after omega-9 enteral injection can further contribute to the already known beneficial properties facilitated by unsaturated fatty acid-enriched diets.

## 1. Introduction

Sepsis is a cause of morbidity and mortality in intensive care units and associated with increased hospital-related costs [1, 2]. According to the Third International Consensus definitions, sepsis is a life-threatening organ dysfunction caused by unbalanced host response to infection [3].

Different strategies for the treatment of sepsis have emerged in the last few years, but none of them has proven to be beneficial in clinical trials [4]. Lipids can modulate leukocyte function and therefore the immune response [5]. The Mediterranean diet, characterized by high ingestion of olive oil, is associated to a reduction in the mortality of vascular diseases and cancer [6–8]. Oleic acid, a *ω*-9 monounsaturated fatty acid, is the main constituent of olive oil [9, 10]. We have previously shown that mice fed with chow rich in olive oil had increased survival rates, decreased neutrophil accumulation, lowered plasma TNF-*α*, prostaglandin E_2_, and leukotriene B_4_ levels in the peritoneal cavity after LPS-induced endotoxic shock [11].

Omega-9 is a natural agonist of peroxisome proliferator-activated receptor (PPAR) [12]. Three PPAR isotypes were described so far: PPAR alpha, PPAR gamma, and PPAR delta/beta. PPARs modulate metabolism, inflammation, and infection [13–15]. PPAR gamma ligands had been demonstrated to protect septic animals against microvascular dysfunction [16] and enhance bacterial elimination through neutrophil extracellular trap formation [17]. Furthermore, we showed that omega-9 decreased nonesterified fatty acids in mice after enteric injection [18] and pretreatment with omega-9 improved lipid metabolism acting on PPAR target genes with increasing survival of septic mice [19].

Here, we investigated the effect of omega-9 on systemic corticosterone levels, inflammatory markers, cell migration, bacterial clearance, and nuclear receptor PPAR gamma expression in both liver and adipose tissues during experimental sepsis. We also studied omega-9 effects on leukocyte rolling *in vivo*.

## 2. Materials and Methods

### 2.1. Animals

Male Swiss mice weighing between 18 and 20 g were obtained from FIOCRUZ (Rio de Janeiro, Brazil) and were purchased from Janvier Lab (Saint Berthevin, France). The animals were accommodated in a room at 22°C, with free access to water and food and alternating light/dark cycle of 12 h. All experiments were approved by the Oswaldo Cruz Foundation Animal Welfare Committee under license number LW-36/10 and L-015/2015 and by the Regierung von Oberbayern, 002-08. The weight of the animals was measured on days 1, 7, and 14, and the food intake was quantified for each cage. We divided the amount of chow that was consumed by the number of animals in each cage, and then, we estimate the food intake per animal.

### 2.2. Omega-9 or Palmitic Acid Treatment

Mice were given a daily dose of omega-9 (oleic acid, 18 : 1 (n-9), Sigma) or palmitic acid (16 : 0, Sigma) for 14 days before CLP. For the intravital microscopy experiments, the animals received omega-9 for 8 days. We prepared oleate solution by water addition according to previous works [20–22]. Briefly, we added NaOH to reach pH 12.0 and sonicated; after oleate solubilization, we adjusted the pH to 7.6 with HCl. We gave by gavage 0.28 mg of omega-9 (100 *μ*L) or 0.26 mg of palmitic acid (100 *μ*L) per day. Control mice received 100 *μ*L of saline.

### 2.3. Cecal Ligation and Puncture (CLP)

Mice received omega-9 or saline for 14 days orally. On the 15^th^ day, we induced polymicrobial sepsis by CLP, as we previously described [19]. Briefly, we anesthetized mice through intraperitoneal injection of ketamine (100 mg/kg) (Cristália) and xylazine (10 mg/kg) (Syntec). We made an incision through the linea alba; the cecum was exposed, ligated with sterile 3-0 silk, and perforated through and through twice with an 18 gauge needle. We extruded a small amount of fecal material through the hole, and the cecum was softly pushed into the abdomen. We sutured the area with nylon 3-0 (Shalon) in two layers. All mice received 1 mL of sterile 0.9% saline subcutaneously. For 24 h experiments, six hours after CLP, we treated mice with antibiotic imipenem (10 mg/kg) intraperitoneally. We submitted sham mice to the same procedures described above, but the cecum was not ligated nor punctured.

### 2.4. Peritoneal Lavage

Mice were submitted to euthanasia with isoflurane (Cristália) 6 h or 24 h after surgery. The peritoneal cavity was washed with saline (3 mL) under sterile conditions. Aliquots from the peritoneal washes were plated in tryptic soy for count of colony forming units (CFU) and used for total cell count in Turk solution (2% acetic acid), in Neubauer chambers. Differential leukocyte count was done in cytocentrifuged smears stained with panoptic (Laborclin). The remaining peritoneal wash was centrifuged, and the supernatant was collected and stored at −20°C for further cytokine quantification. We also counted total leukocytes in blood samples taken from a tail vein and analyzed differential leukocyte counts in blood smears.

### 2.5. Cytokine Analysis

TNF-*α*, IL-10, and IL-1*β* were detected by enzyme-linked immunosorbent assay (ELISA, DuoSet kit, R&D systems, Minneapolis, MN, USA) according to the instructions of the manufacturer.

### 2.6. Western Blot Analysis

Detection of PPAR gamma was performed as previously described [22] with minor modifications. Briefly, we perfused organs with 20 mM ethylenediaminetetraacetic acid (EDTA) pH 7.4. We cut liver tissues into small pieces and mixed with lysis buffer (with a cocktail of protease inhibitors) at 4°C in (Complete, Roche AG, Basel, Switzerland). We lysed periepididymal adipose tissues at 4°C in RIPA buffer with protease inhibitors (Roche AG, Basel, Switzerland) and phosphatase inhibitor cocktail (Roche). We stored tissues at −20°C for further protein quantification by BCA. Western blot analysis was done with whole liver and adipose tissue lysates (40 *μ*g of proteins) using anti-PPAR gamma (1 : 1000, Santa Cruz) and anti-*β*-actin (1 : 15000 dilution, Sigma), and detection was performed with the “SuperSignal Chemiluminescence” kit (Pierce), after exposing the membrane to an autoradiograph film (GE Healthcare). Bands were digitalized and quantified by the ImageMaster 2D Elite program.

### 2.7. Corticosterone Levels

Animals were euthanized using an overdose of isoflurane (Cristália), during the nadir (08:00 h) of the circadian rhythm [23, 24], and blood was straightway collected through cardiac puncture with saline with heparin (400 U/mL). Plasma was obtained after sample centrifugation for 10 min at 1000 ×g and stored at −20°C until use. Corticosterone plasma levels were evaluated using radioimmunoassay (MP Biomedicals, Solon, OH, USA) following the guidelines of the manufacturer.

### 2.8. Intravital Microscopy of the Cremaster Muscle

Intravital microscopy of the mouse cremaster muscle postcapillary venules was used to study leukocyte rolling under different inflammatory conditions as previously described [25]. Briefly, we anesthetized the animals with intraperitoneal injection of ketamine (125 mg/kg, Ketanest®, Pfizer GmbH, Karlsruhe, Germany) and xylazine (12.5 mg/kg; Rompun®, Bayer, Leverkusen, Germany). Afterward, they were transferred the animals to a heating pad to keep temperature at 37°C. After surgical insertion of a tracheal tube, the carotid artery was cannulated to take the blood sample and for systemic application of antibodies. We used the P-selectin-blocking mAb RB40.34 and the E-selectin-blocking mAb 9A9 which were generous gifts from Dietmar Vestweber (MPI Münster) and Barry Wolitzky (MitoKor, San Diego), respectively. The scrotum was surgically opened, to exteriorize the cremaster muscle. After longitudinal incision and distribution of the muscle over a cover glass, the cremaster muscle was superfused with 35°C bicarbonate-buffered saline. We observed cremaster muscle postcapillary venules via an upright microscope (Olympus BX51) with an objective (×40/0.8 NA). We measured venular centerline red blood cell velocity during the experiment via an online cross-correlation program (CircuSoft Instrumentation, Hockessin, Delaware, USA). We recorded the experiments via a CCD camera system (model CF8/1; Kappa, Gleichen, Germany) on a Panasonic S-VHS recorder and performed offline the analysis of experiments using the used videotapes. We measured diameter and segment length of postcapillary venules using a digital image processing system [26]. Postcapillary venules were recorded to calculate rolling flux fraction (percentage of rolling leukocytes relative to the number of leukocytes passing the vessel). Leukocytes with a displacement of >15 *μ*m were tracked by using ImageJ (National Institutes of Health, Bethesda, MD). In some experiments, TNF-*α* (500 ng) was injected intrascrotally 2.5 h before intravital imaging.

### 2.9. Intravital Microscopy of Brain Microcirculation

The cerebral microcirculation in mice was assessed as previously described [16]. Briefly, we anesthetized the animals with ketamine (75 mg/kg, i.p.) and xylazine (10 mg/kg, i.p.) and fixed in a stereotaxic frame. Then, the left parietal bone was exposed by a midline skin incision; a cranial window overlying the right parietal bone (1–5 mm lateral, between the coronal suture and the lambdoid suture) was created with a high-speed drill, and the dura mater and the arachnoid membranes were excised and withdrawn to expose the cerebral microcirculation. The cranial window was suffused with artificial cerebrospinal fluid (in mmol: KCl, 2.95; NaCl, 132, CaCl_2_, 1.71; MgCl_2_, 0.64; NaHCO_3_, 24.6; dextrose, 3.71; and urea, 6.7; at 37°C, pH 7.4). Animals were then placed under an upright fixed-stage intravital microscope equipped with a LED lamp (Zeiss, model Axio Scope) coupled to a Zeiss Axiocam and processed using ZEN software (Zeiss). Water immersion objective 20x were used in the experiments and produced total magnifications of 200x.

The visualization of brain microvascular surface was facilitated by intravenous administration of 0.1 mL 2% fluorescein isothiocyanate- (FITC-) labeled dextran (molecular weight 150,000) and by epi-illumination at 460–490 nm using a 520 nm emission filter. Leukocytes were labeled using the fluorescent dye rhodamine 6G (0.3 mg/kg) and visualized by epi-illumination at 536–556 nm excitation using a 615 nm emission wavelength. Analysis of leukocyte-endothelium interactions was carried out by analyzing four randomly selected venular segments (30 to 100 mm in diameter) in each preparation. Rolling leukocytes were counted as the number of cells crossing the venular segment at speed less than the red blood cells for 1 minute. Adherent leukocytes were defined as the total number of leukocytes that were firmly attached to the endothelium and did not change position during 1 minute of observation and expressed as a number of cells/mm^2^/100 *μ*m.

### 2.10. Statistical Analysis

Results were analyzed by “one-way” ANOVA followed by Newman-Keuls using GraphPad Prism 5.0. Values of *p* < 0.05 were considered significant. Data are presented as mean ± SEM or individual values with a median.

## 3. Results

### 3.1. Omega-9 Treatment Decreased Corticosterone Serum Levels in Septic Mice

We previously demonstrated that omega-9 treatment increased survival and ameliorated clinical scores after CLP-induced sepsis [19]. In the present work, we continue to investigate the mechanisms behind the protective effects of omega-9.

**Figure 1:**
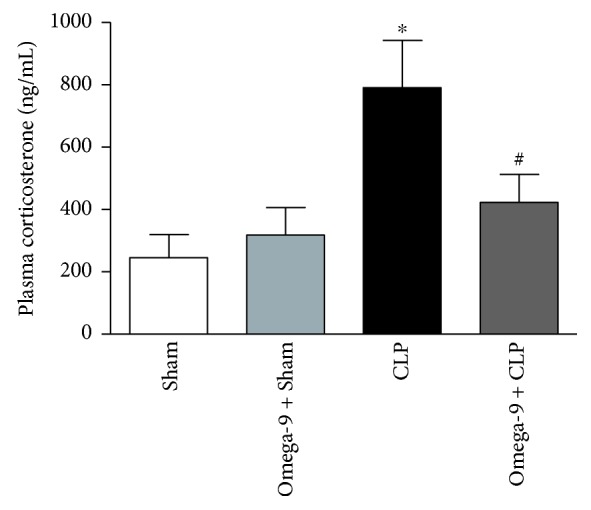
Omega-9 decreased cortisol levels in septic mouse plasma. Animals were treated with omega-9 for 14 days. On the 15^th^ day, CLP was performed, and 24 h after, the plasma was collected for the quantification corticosterone. Each bar represents the mean *±* SEM of at least 7 animals. ∗ and + *p* < 0.05 compared to sham and sham + omega-9, respectively, and # compared to CLP.

High cortisol levels (a 10-fold increase compared to health voluntaries) [27] are linked to disease severity and hyperinflammation during sepsis. Here, we observed high levels of corticosterone in septic mice. Omega-9 treatment prevented the increase in plasma corticosterone levels (Figure 1), reinforcing our previous data where omega-9 pretreatment decreased biochemical markers of organ dysfunction [19].

### 3.2. Omega-9 Reduced IL-1*β* and TNF-*α* and Increased IL-10 Production in Septic Mice

**Figure 2:**
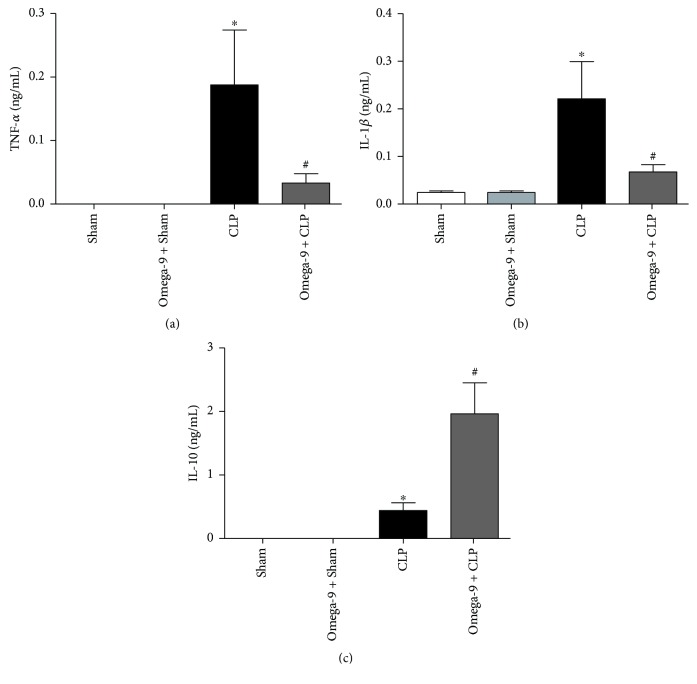
Omega-9 reduced proinflammatory cytokines but increases the level of the anti-inflammatory cytokine IL-10 in the peritoneal lavage of mice submitted to CLP. Animals were treated with omega-9 for 14 days. On the 15^th^ day, CLP was performed, and 24 h after, the peritoneal lavage was collected for the quantification of TNF-*α* (a), IL-1*β* (b), and IL-10 (c). Each bar represents the mean *±* SEM of at least 7 animals. ∗ and + *p* < 0.05 compared to sham and sham + omega-9, respectively, and # compared to CLP.

Monocytes and neutrophils produce IL-1*β*, tumor necrosis factor-*α* (TNF-*α*), and IL-10, cytokines constituting the storm during sepsis [27–29]. Septic mice had higher levels of TNF-*α*, IL-*β*, and IL-10 in the peritoneal lavage compared to the control (Figures 2(a)–2(c)) while omega-9 pretreatment strongly decreased the levels of TNF-*α* (Figure 2(a)) and IL-1*β* (Figure 2(b)) in septic mice. Interestingly, IL-10 increased in the peritoneal lavage of septic mice receiving omega-9 (Figure 2(c)).

### 3.3. Omega-9 Decreased Neutrophil Migration in the Peritoneum of Septic Mice

**Figure 3:**
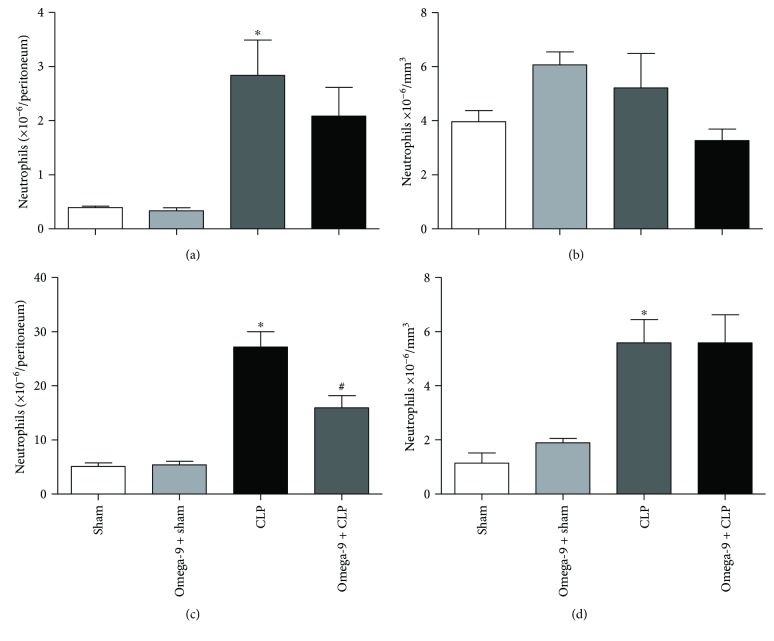
Omega-9 reduced leukocyte migration to the peritoneal cavity in septic mice. Animals were treated with omega-9 for 14 days. On the 15^th^ day, CLP was performed; 6 h and 24 h after, the peritoneal lavage was collected for the leukocyte counts. Counts of peritoneal neutrophils (a) and systemic neutrophils (b) 6 h after CLP and counts of peritoneal neutrophils (c) and systemic neutrophils (d). Control groups received the same volume of saline. Results are mean ± SEM from at least 7 animals. ∗ and + *p* < 0.05 compared to sham and sham + omega-9, respectively, and # compared to CLP.

One of the main steps of the immune response during inflammation is the recruitment of myeloid cells into inflamed tissue. We evaluated the effect of omega-9 pretreatment on cell migration and accumulation into the peritoneal cavity of septic mice. Septic mice presented higher leukocyte numbers in the peritoneal cavity, characterized by an increase in neutrophil numbers when compared to sham animals in both time points analyzed, 6 h and 24 h after CLP (Figures 3(a) and 3(c)). Neutrophil accumulation in the peritoneal cavity was reduced in septic mice treated with omega-9 only 24 h after CLP (Figure 3(c)), showing no significant effect at an earlier time point (Figure 3(a)).

### 3.4. Omega-9 Impaired Leukocyte Rolling in Inflamed Microvessels *In Vivo*

**Figure 4:**
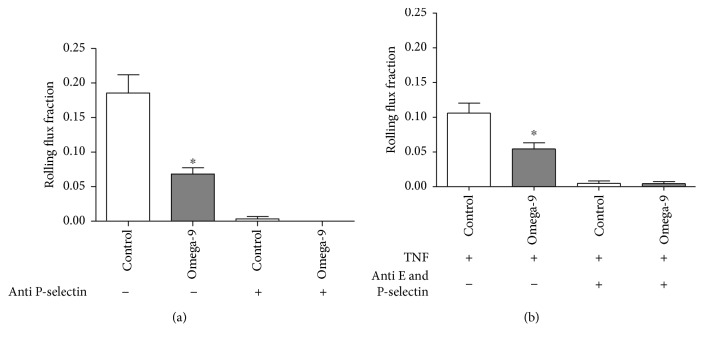
Omega-9 reduced rolling flux fraction in cremaster in trauma and TNF models. We treated the animals with omega-9 for 8 days prior to the experiments. On the day 9, we analyzed rolling in postcapillary venules of mouse cremaster muscle in two models: trauma (a) and TNF (b) models. We also treated animals of the trauma model with anti-P-selectin and of the TNF model with anti-P- and E-selectins. Rolling flux fraction was analyzed. Each bar is mean from at least 5 animals. ∗ and + *p* < 0.05 compared to sham and sham + omega-9, respectively, and # compared to CLP.

To analyze the role of omega-9 in leukocyte rolling *in vivo*, we used intravital microscopy in surgically prepared mouse cremaster muscle postcapillary venules [30, 31]. Leukocyte rolling is induced by the surgery of the cremaster and is exclusively dependent on P-selectin (<45 min after surgery) [32–35]. We showed a decrease in rolling in omega-9-treated mice compared to the control (Figure 4(a) and Supplemental Movies 2 and 1, respectively). Systemic injection of P-selectin-blocking antibody RB40.34 abolished leukocyte rolling (Figure 4(a)) endorsing the dependence of P-selectin on rolling in the trauma model. Next, we used TNF-*α* stimulation of the mouse cremaster muscle in Swiss mice pretreated with omega-9 to study leukocyte rolling. In TNF-*α*-stimulated mice, leukocyte rolling is P- and E-selectin dependent [35]. We found that rolling flux fraction was significantly diminished in omega-9-treated animals compared to that in controls (Figure 4(b)). There was no alteration in neutrophil blood counts comparing omega-9-treated and untreated animals (data not shown). Microvascular injection of anti-P-selectin and anti-E-selectin-blocking antibodies Rb40.34 and 9A9, respectively, abolished rolling completely demonstrating that rolling in this model is indeed dependent on P- and E-selectins, as shown previously [35]. Hemodynamic conditions were alike between the different treatment groups (Supplemental Table 1).

### 3.5. Omega-9 Impaired Leukocyte Rolling in Septic Mice

**Figure 5:**
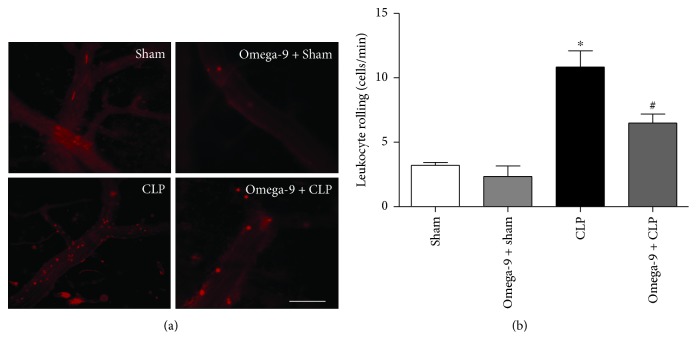
Omega-9 reduced leukocyte rolling in mice submitted to CLP. Fluorescent intravital microscopy images showing leukocyte-endothelium interaction in the cerebral postcapillary venules after 24 h of sepsis in mice (a). Animals were treated with vehicle (CLP) or omega-9 (omega-9 + CLP) compared to sham operated pretreated with vehicle (sham) or omega-9 (omega-9 + sham) mice. Rolling of leukocytes in the microvasculature was expressed as number of cells per minute (b). Data indicate mean ± SEM, 5 mice per group. ^∗^ *p* < 0.001 versus the sham group; ^#^ *p* < 0.05 versus the CLP group.

Figure 5 illustrates the leukocyte-endothelium interaction in cerebral venules of mice subjected to sham or CLP with (omega-9 + CLP) or without (CLP) omega-9 treatment. Rolling leukocytes in the CLP group were significantly increased when compared to the sham group. Pretreatment with omega-9 significantly attenuated the CLP-induced leukocyte rolling in the cerebral microcirculation compared with the CLP-untreated group. Omega-9 pretreatment did not induce any effect in cerebral venules of sham mice.

### 3.6. Omega-9 Increased Bacterial Killing in the Mouse Peritoneal Cavity after CLP

**Figure 6:**
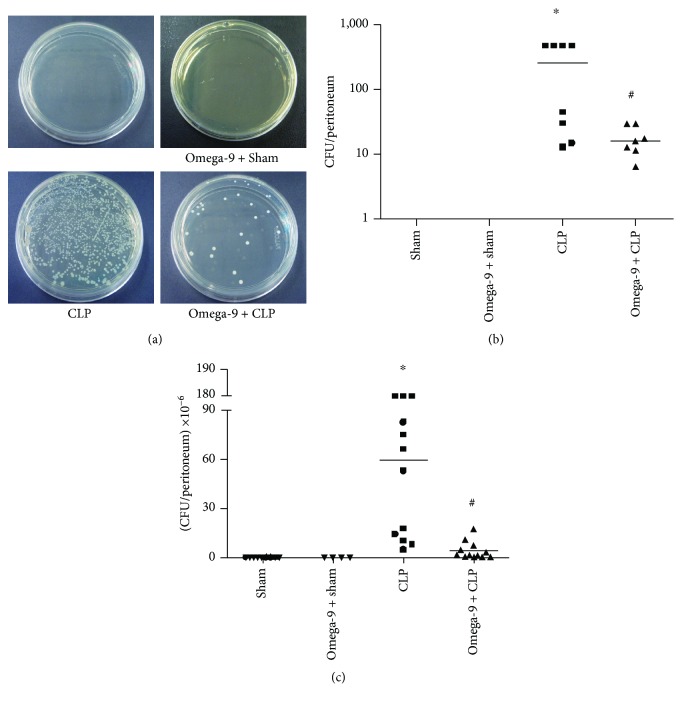
Omega-9 improved bacterial clearance in Swiss mice submitted to CLP. Animals were treated with omega-9 for 14 days. On the 15^th^ day, CLP was performed, and 6 h (b) and 24 h (c) after, the peritoneal lavage was collected and plated on TSA-coated plates for CFU counts. Results are represented as individual values and median from at least 7 animals. In (a), there are representative photos of the exposed graph (c). ∗ and + compared to sham and sham + omega-9, respectively, and # compared to CLP.

Because omega-9 treatment modulated cytokine response and neutrophil accumulation in the peritoneal cavity, we decided to evaluate the impact of omega-9 treatment on the bacterial load after CLP. We observed that despite decreasing neutrophil accumulation in the peritoneal cavity, omega-9 pretreatment did not impair the bacterial elimination by the innate immune response. To our surprise, omega-9 pretreatment increased bacterial clearance in the peritoneum (Figure 6).

### 3.7. Omega-9 Restored PPAR Gamma Expression in the Liver and Adipose Tissue in Septic Mice

**Figure 7:**
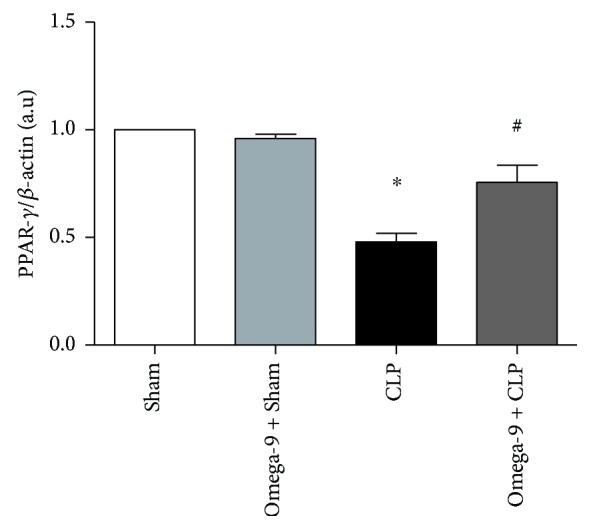
Omega-9 treatment restored the expression of PPAR gamma in the liver of CLP mice. Animals were treated with omega-9 for 14 days. On the 15^th^ day, CLP was performed, and liver was removed from animals 24 h after CLP. Graphics in this figure represent the rate between densitometric analyses of PPAR gamma and *β*-actin bands. ∗ and + *p* < 0.05 compared to sham and sham + omega-9, respectively, and # compared to CLP.

**Figure 8:**
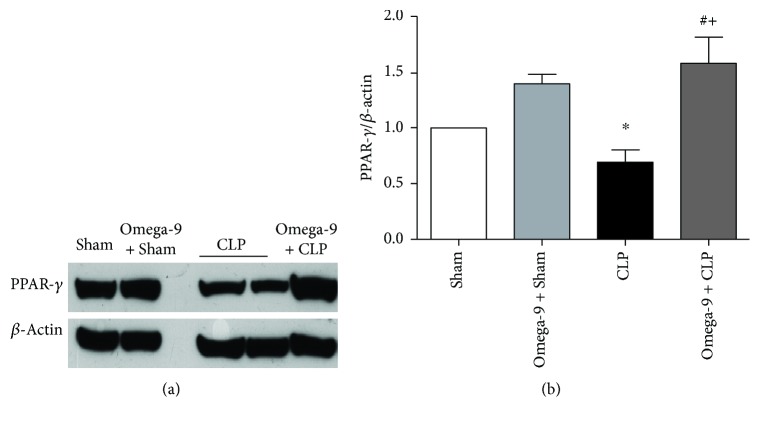
Omega-9 treatment restored PPAR gamma expression in the adipose tissue of CLP mice. Animals were treated with omega-9 for 14 days. On the 15^th^ day, CLP was performed, and adipose tissue was removed from animals 24 h after CLP. Graphics in this figure represent the rate between densitometric analyses of PPAR gamma and *β*-actin bands. ∗ and + *p* < 0.05 compared to sham and sham + omega-9, respectively, and # compared to CLP.

Omega-9 is a PPAR ligand and the treatment displayed an anti-inflammatory profile, so we investigate the levels of PPAR gamma in the liver and adipose tissues. We confirmed the reduction of PPAR gamma expression in both liver and adipose tissues from septic mice. In contrast, omega-9-pretreated animals maintained PPAR gamma expression levels similar to sham mice in the liver (Figure 7), and it was even higher in adipose tissue (Figure 8).

## 4. Discussion

Mortality from sepsis varies from 30 to 50%, and incidences are rising due to a rising elderly population and an increased number of patients with immunosuppression [36–39]. The number of patients with sepsis rose from 387,330 to 1.1 million from 1996 to 2011 and probably will reach 2 million by 2020 in the US [40]. Sepsis mortality is similar to heart attacks and exceeds stroke deaths. Therapeutic procedures are urgently needed [41]. Hence, infections leading to damage in the microcirculation can compromise the multiple organ function, including the lungs, heart, liver, gut, kidneys, and brain, causing hypotension and myocardial dysfunction, microvascular leak, thrombocytopenia, disseminated intravascular coagulation (DIC), acute respiratory distress syndrome (ARDS), acute kidney injury (AKI), and acute brain injury [42–45].

Therapies with anti-Toll-like receptor 4, anti-TNF-*α*, and activated protein C failed in clinical trials, requiring a rethinking of sepsis pathophysiology [29, 46–51]. Food intake can influence the immune response [52–57]. The Mediterranean diet, composed of olive oil as the main source of fat, is an example of how lipids can influence the inflammatory response [7, 58]. This diet has been linked with a reduced risk of cancer and vascular illnesses and also with a decreased chronic disease incidence, such as Parkinson [6, 8, 59]. Omega-9 is a monounsaturated fatty acid, the main olive oil component [9, 10]. Omega-9 protects from insulin resistance and prevents endothelial dysfunction in response to proinflammatory signals. Omega-9 also reduces vascular smooth muscle cell proliferation and apoptosis, suggesting a beneficial role in atherosclerosis [60]. Furthermore, omega-9 decreases the release of cytokines, increases the killing ability of neutrophils, and improves bacterial elimination [61].

We previously have shown that omega-9 prevents organ dysfunction and increases survival during sepsis [19]. Some reports associated kidney and liver dysfunction during sepsis with an increase in plasma cortisol levels and decreased ability to metabolize cortisol. Both the adrenal gland activation to produce glucocorticoid and catecholamine and the diminished ability to break down cortisol by suppressed expression and impaired cortisol-metabolizing enzyme activity are characteristic of host innate reaction to aggression [62, 63]. Here, we showed that septic animals had increased corticosterone plasma levels which could be decreased by omega-9 treatment. We suggest that omega-9 protective effect on organ dysfunction may be at least partially related to its effect on normalizing corticosterone levels in our animal model.

Neutrophils are the highest leukocyte population in the blood of humans (50–70% of leukocytes). They can be quickly mobilized from bone marrow into the circulation after immune activation and physical exercise or caused by the release of corticoids and adrenaline [64]. Reservoir organs may contribute to fast mobilization during inflammatory processes. Recruitment of neutrophil to the inflammatory site is a process that comprehends tethering, rolling, adhesion, crawling, and extravasation. A spatial and temporal expression and adhesion molecule interaction on neutrophils (i.e., L-selectin, PSGL-1, LFA-1, and Mac-1) and their ligands on endothelial cells (i.e., E- and P-selectin, and ICAM-I) are crucial for effective extravasation of neutrophils into the tissues [65]. Negatively modulating the expression of these adhesion molecules, in turn, will influence leukocyte migration into the inflamed tissue.

Ingestion of the monounsaturated fatty acid-rich diet decreased the expression of ICAM-I [66]. Human embryonic endothelial cells (HUVECs) treated with omega-9 had diminished expression of LPS-stimulated VCAM-I, E-selectin, and ICAM-I [67]. Mice fed with chow rich in olive oil decreased neutrophil accumulation in the peritoneal cavity 24 h after LPS injection [11]. Our data add to this as they showed a decrease of neutrophil influx into the peritoneum in omega-9-treated animals, preventing exacerbated inflammation. Interestingly, just the treatment with unsaturated fatty acid was effective in controlling neutrophil influx, because supplementation with a saturated fatty acid palmitic acid did not affect neutrophil accumulation in septic mice (Supplemental Figure 1). Using the trauma model, where rolling depends on P-selectin, omega-9-treated animals showed a decrease in rolling that could be confirmed in the TNF model suggesting that omega-9 regulates selectin-dependent rolling *in vivo*. Omega-9 effect reducing leukocyte rolling extended to septic animals. In the sepsis model, omega-9 was also very effective in inhibiting rolling of leukocytes on endothelial cells of septic mice.

Neutrophils fight and destroy invading microorganisms by diverse mechanisms such as phagocytosis, production of ROS, and formation of neutrophil extracellular trap (NET) [68]. Neutrophils produce proinflammatory cytokines and release nitric oxide and ROS [69], and the excess of these mediators can increase vascular permeability leading to organ damage [70, 71]. By attenuating the accumulation of neutrophils in the peritoneum, there is a decrease in organ damage caused by the excessive overactivated neutrophil numbers. Interestingly, we also detected increased bacterial clearance in the peritoneal lavage in omega-9-treated septic animals. Similar results have been obtained recently by our group using low dose dasatinib treatment in septic mice [72]. Neutrophils increase their ability to produce ROS after treatment with omega-9 [73]. Supplementation for only 5 days is enough for omega-9 to incorporate into neutrophil membranes [74]. Also, omega-9 enhanced phagocytosis by neutrophils 30 min after incubation and improved the microorganism elimination *in vitro* [75]. These effects are not achieved using omega 3 or 6 [65]. Our results showed that omega-9 was effective in increasing the bacterial elimination by the host during sepsis. Intake of omega-3 daily for 14 days alters gut flora decreasing species diversity, but several butyrate-producing bacteria increased [76]. Similarly, a decrease in Faecalibacterium, often linked to an increase in the Bacteroidetes and butyrate-producing bacteria belonging to the Lachnospiraceae, has been observed following omega-3 supplementation [77]. Accordingly, a study suggests that PUFA supplementation improves gut function and microbiome composition [78]. Concerning infection models, neutrophils treated with omega-3 showed enhanced antiparasitic activity against *Plasmodium falciparum* [79] and dietary omega-3 decreased bacterial load and increased the survival rate in septic mice [80]. In our sepsis model, it is possible that although there were fewer neutrophils in the peritoneum, they are still able to fight the infection efficiently, actively killing bacteria without causing excessive tissue damage. Omega-9 just prevented excessive neutrophil influx, because it did not affect early neutrophil migration to the peritoneal cavity but inhibited exacerbated neutrophil influx 24 h after CLP. CFU counts corroborate because bacterial elimination was effective even in earlier time point in omega-9-treated septic animals. We do not exclude the macrophage role on bacterial killing. So far, our data only allow us to conclude that omega-9 has not altered mononuclear cell counts (data not shown).

Omega-9 can bind to PPAR, known as lipid sensors [12]. PPAR are ligand-activated transcription factors with an important role in the inflammation and lipid and glucose metabolism [13, 14]. PPAR gamma activation diminished inflammatory response, increased survival, and attenuated neutrophil migration in different models of inflammation [81–84]. Moreover, we have shown that mice fed with omega-9 and submitted to sepsis produced less proinflammatory cytokines and more IL-10, which agrees with studies showing the activation of PPAR gamma-enhanced production of the anti-inflammatory cytokine IL-10 [17, 85, 86]. We showed that PPAR gamma decreases rolling and adhesion in brain microcirculation of septic mice [16]. Literature shows endothelial PPAR gamma downregulates P-selectin expression decreasing leukocyte-endothelial interactions [87]. Omega-9 binding to PPAR gamma may modulate P-selectin expression on leukocytes, decreasing their ability to roll. PPAR also increases bacterial elimination. Lack of PPAR alpha is linked with a high bacterial load in septic mice [88]. We showed that PPAR gamma rosiglitazone leads to increased bacterial clearance in septic mice. Leukocytes from PPAR gamma agonist-treated septic animals are activated; they increased intracellular ROS and increased the capacity of killing bacteria by NET formation [17].

PPAR gamma expression is decreased in many organs like lung, liver, and adipose tissue during endotoxemia and sepsis [81, 89]. Interestingly, endotoxin decreased PPAR gamma through the increase of TNF release [90]. Based on the findings by Zhou et al. and our own results, we suggested the correlation between TNF production and decreased PPAR gamma expression. Studies with phytochemical curcumin have related its anti-inflammatory potential and mortality protection to increased PPAR gamma expression in the liver [91]. Our data showed that PPAR gamma expression in the liver decreases in septic animals and omega-9 treatment increases it, suggesting that PPAR gamma liver expression may be involved in omega-9-protective effects during sepsis.

Adipose tissue plays an essential role on the inflammatory response regulation in many metabolic diseases, including metabolic syndrome, obesity, diabetes, and sepsis [92, 93]. PPAR gamma controls adipocyte differentiation and function. LPS or TNF alpha decreased PPAR gamma expression in adipose tissue [94], as seen in our model of sepsis. The capacity of maintaining the anti-inflammatory grade of visceral adipose tissue by the PPAR gamma agonist is associated with the prevention of lung injury observed during sepsis. The PPAR gamma agonist pioglitazone decreased mortality of septic mice because it diminished inflammatory cytokine production in omental tissue, controlling visceral adipose tissue inflammation [93]. We reinforce the role of adipose tissue in negative modulation of exacerbated inflammation during sepsis. PPAR gamma expression in adipose tissue may be relevant because it was lower in septic animals and it was restored by omega-9 treatment. PPAR gamma expression is induced by its ligands (Frygiel-Górniak, 2014). Although omega-9 has other targets, we believe that omega-9 binding to PPAR gamma would restore PPAR gamma protein expression and account, at least partially, for omega-9-modulatory effect during sepsis.

In our previous report, we showed that omega-9 improves lipid metabolism in septic mice increasing their survival by activating PPAR-regulated genes [19]. Accordingly, herein, we showed that omega-9 treatment dampens inflammation and increases bacterial clearance in septic mice possibly involving PPAR gamma. Therefore, omega-9 treatment has dual effect regulating lipid metabolism and inflammation.

### 4.1. Conclusion and Consideration

Omega-9 modulated the immune response in septic mice. Omega-9 decreased the production of proinflammatory cytokines, increased IL-10 production, reduced neutrophil migration and accumulation in the site of infection, and also improved bacterial clearance. Omega-9 treatment affected leukocyte trafficking in septic animals and in inflamed cremaster muscle postcapillary venules by decreasing selectin-dependent leukocyte rolling *in vivo*. Those effects controlling inflammation and increasing bacterial clearance likely contribute to the better outcome of sepsis. Therefore, omega-9-enriched diet, particularly olive oil, as supplemental food, may be advisable in patients with infections and might sum up with the other benefits of the ingestion of diets composed of unsaturated fatty acids.

## Data Availability

All data used to support the findings of this work are included within the article and the supplementary information file.

## Supplementary Materials

Supplementary 1 Supplemental videos from intravital microscopy in surgically prepared mouse cremaster muscle postcapillary venules in the trauma model are available. Video 1 was recorded from untreated animals and video 2 from the omega-9-treated group.  Figure 4 shows the data.

Supplementary 2 Supplemental Table 1: hemodynamic conditions and systemic leukocyte counts from untreated or omega-9-treated Swiss mice in trauma and TNF models. We also treated animals of the trauma model with anti-P-selectin and of the TNF model with anti-P- and E-selectins.

Supplementary 3 Supplemental Figure 1: palmitic acid effect on neutrophil accumulation in the peritoneal cavity in septic mice. Animals were treated with palmitic acid for 14 days. On the 15^th^ day, CLP was performed, and 24 h after, the peritoneal lavage was collected for the neutrophil counts. Control groups received saline. Results are mean ± SEM from at least 6 animals. The experiment was repeated twice. ^∗^ *p* < 0.05 compared to sham and sham + palmitic acid.
