# Supplementary material for: Omega-9 Oleic Acid, the Main Compound of Olive Oil, Mitigates Inflammation during Experimental Sepsis
Source: Oxid Med Cell Longev. 2018 Nov 13;2018:6053492. doi: 10.1155/2018/6053492 (PMC6260523; doi:10.1155/2018/6053492)
Supplement: Supplementary 2 — Supplemental Table 1: hemodynamic conditions and systemic leukocyte counts from untreated or omega-9-treated Swiss mice in trauma and TNF models. We also treated animals of the trauma model with anti-P-selectin and of the TNF model with anti-P- and E-selectins. [file 6053492.f2.docx]

Supplemental table 1

|  | |  |  | | Average | | | | |
| --- | --- | --- | --- | --- | --- | --- | --- | --- | --- |
| Model | Treatment | | | animals | Venules | Diameter (µm) | Centerline Velocity (µm/s) | Shear rate  (s^-1^) | Systemic leukocyte count (cell/micro) |
| Trauma | Oleic acid | | | 4 | 22 | 31,7 | 2196 | 1761,9 | 6275 |
|  | Pure water | | | 4 | 17 | 31,6 | 2302 | 1789,5 | 5996 |
|  | Oleic acid + Anti P-sel | | | 3 | 9 | 32,8 | 2478 | 1950 | 6616 |
|  | Pure water + Anti P-Sel | | | 3 | 9 | 33,7 | 3133 | 2288 | 5750 |
| TNFα | Oleic acid | | | 4 | 19 | 34,4 | 1648 | 1185,9 | 2905 |
|  | Pure water | | | 2 | 10 | 33,1 | 2210 | 1660,1 | 2495 |
|  | Oleic acid + Anti E and P-sel | | | 4 | 11 | 35,25 | 1545 | 1098 | 5503 |
|  | Pure water + Anti E and P-sel | | | 2 | 6 | 32,8 | 4500 | 3627 | 6275 |
