# Supplementary material for: Omega-9 Oleic Acid, the Main Compound of Olive Oil, Mitigates Inflammation during Experimental Sepsis
Source: Oxid Med Cell Longev. 2018 Nov 13;2018:6053492. doi: 10.1155/2018/6053492 (PMC6260523; doi:10.1155/2018/6053492)
Supplement: Supplementary 3 — Supplemental Figure 1: palmitic acid effect on neutrophil accumulation in the peritoneal cavity in septic mice. Animals were treated with palmitic acid for 14 days. On the 15th day, CLP was performed, and 24 h after, the peritoneal lavage was collected for the neutrophil counts. Control groups received saline. Results are mean ± SEM from at least 6 animals. The experiment was repeated twice. ∗ p < 0.05 compared to sham and sham + palmitic acid. [file 6053492.f3.docx]

Supplemental figure 1
